# A Comprehensive Review on Bariatric Endoscopy: Where We Are Now and Where We Are Going

**DOI:** 10.3390/medicina59030636

**Published:** 2023-03-22

**Authors:** Aurelio Mauro, Francesca Lusetti, Davide Scalvini, Marco Bardone, Federico De Grazia, Stefano Mazza, Lodovica Pozzi, Valentina Ravetta, Laura Rovedatti, Carmelo Sgarlata, Elena Strada, Francesca Torello Viera, Letizia Veronese, Daniel Enrique Olivo Romero, Andrea Anderloni

**Affiliations:** 1 Gastroenterology and Endoscopy Unit, Fondazione IRCCS Policlinico San Matteo, 27100 Pavia, Italy; 2 Specialization School of Diseases of Digestive System Pavia, University of Pavia, 27100 Pavia, Italy; 3 Digestive Endoscopy Unit, Hospital Nacional Zacamil, San Salvador 01120, El Salvador

**Keywords:** endoscopic bariatric therapy, obesity, sleeve gastrectomy, intragastric balloons, endoscopic sleeve gastrectomy, POSE, transoral outlet reduction, duodenal mucosal resurfacing

## Abstract

*Background:* Obesity is a chronic disease that impairs quality of life and leads to several comorbidities. When conservative therapies fail, bariatric surgical options such as Roux-en-Y gastric bypass (RYGB) and sleeve gastrectomy (SG) are the most effective therapies to induce persistent weight loss. Over the last two decades, bariatric endoscopy has become a valid alternative to surgery in specific settings. *Primary bariatric endoscopic therapies:* Restrictive gastric procedures, such as intragastric balloons (IGBs) and endoscopic gastroplasty, have been shown to be effective in inducing weight loss compared to diet modifications alone. Endoscopic gastroplasty is usually superior to IGBs in maintaining weight loss in the long-term period, whereas IGBs have an established role as a bridge-to-surgery approach in severely obese patients. IGBs in a minority of patients could be poorly tolerated and require early removal. More recently, novel endoscopic systems have been developed with the combined purpose of inducing weight loss and improving metabolic conditions. Duodenal mucosal resurfacing demonstrated efficacy in this field in its early trials: significant reduction from baseline of HbA1c values and a modest reduction of body weight were observed. Other endoscopic malabsorptive have been developed but need more evidence. For example, a pivotal trial on duodenojejunal bypasses was stopped due to the high rate of severe adverse events (hepatic abscesses). Optimization of these more recent malabsorptive endoscopic procedures could expand the plethora of bariatric patients that could be treated with the intention of improving their metabolic conditions. *Revisional bariatric therapies:* Weight regain may occur in up to one third of patients after bariatric surgery. Different endoscopic procedures are currently performed after both RYGB and SG in order to modulate post-surgical anatomy. The application of argon plasma coagulation associated with endoscopic full-thickness suturing systems (APC-TORe) and Re-EndoSleeve have shown to be the most effective endoscopic treatments after RYGB and SG, respectively. Both procedures are usually well tolerated and have a very low risk of stricture. However, APC-TORe may sometimes require more than one session to obtain adequate final results. The aim of this review is to explore all the currently available primary and revisional endoscopic bariatric therapies focusing on their efficacy and safety and their potential application in clinical practice.

## 1. Introduction

Obesity is defined as a body mass index (BMI) equal to or higher than 30 kg/m^2^. It is a pandemic disease that affects 650 million people throughout the world with a continuously increasing incidence [1,2]. 

Genetic predisposition, unbalanced long-term diets, and sedentary habits are the main factors contributing to the multifactorial etiology of this disease. Numerous illnesses result from obesity, mainly type 2 diabetes, arterial hypertension, liver steatosis, and other cardiovascular complications [3]. Moreover, the social implications of this disease significantly hamper everyday activities, contributing to the reduced quality of life of bariatric patients [4]. 

The first-line treatment is based on diet regimens and modifications of lifestyle habits to increase physical activities. In order to obtain valid results, patients’ compliance is essential due to the long-lasting challenging process required [5]. However, these options are frequently insufficient to reach adequate weight loss, and associated treatments are necessary. Pharmacotherapy with medications mainly promoting satiety [3] can be associated with dietary measures, but their efficacy is limited, and their various side effects limit long-term use [6]. Therefore, bariatric surgery has gained relevance in the field, becoming by far the most effective and durable option for obesity treatment. Different types of bariatric surgery have been developed over the years, among which Roux-en-Y gastric bypass (RYGB) and Sleeve gastrectomy (SG) are the most frequently performed [3]. Different from medical and dietary approaches, bariatric surgery allows significant results in terms of weight loss, up to 25–30% from basal weight, even during the long-term period [7,8]. 

The efficacy and safety of bariatric surgery are currently consolidated by a large number of publications [9,10]. As far as the safety profile is concerned, perioperative mortality has dramatically improved since the early 2000s [10,11]. A recent meta-analysis showed an early (<30 days) major adverse events rate of 0–1.6% with a mortality rate of 0–0.6% [12]. However, since obesity is continuously increasing in prevalence [2] and patients addressed to bariatric surgery are often young and/or fragile with multiple comorbidities, less invasive and safer options are desirable to treat this challenging benign disease. 

With this purpose, over the years, bariatric endoscopic procedures have been developed in order to offer less invasive options, with an expected total body weight loss of at least 10–20% [13] when associated with appropriate dietary restrictions [14,15,16]. They can be classified as primary treatments or revisional procedures after surgery failure. 

Primary bariatric endoscopic treatments, which are currently approved by the Food and Drug Administration (FDA) or the European Community (CE), work by reducing gastric volume by means of specific devices. The reduction of gastric volume can be either achieved by placing space-occupying devices or by creating an endoscopic gastroplasty, plicating the stomach walls in order to decrease gastric lumen [14]. In recent years, new endoscopic devices and procedures that reduce the contact between food and the gastrointestinal wall have been developed, with the aim of mimicking surgical malabsorptive procedures [17,18,19]. One of the main goals of these novel endoscopic treatments is to combine the weight loss effect with an improvement of metabolic complications that are the most common cause of morbidity in obese patients. Revisional bariatric endoscopic procedures have been developed to maximize patients’ outcomes after surgical treatments [15]. Indeed, weight regain after bariatric surgery potentially occurs in one third of patients, and re-surgery in this category of patients is characterized by a high rate of complications [20]. After RYGB, dilation of the gastro-jejunal anastomosis and of the gastric pouch may occur, reducing the satiety sensation. Similarly, afterward, SG, dilation of the gastric remnant could lead to weight regain [15]. Endoscopic bariatric revisional procedures are aimed to have a restrictive effect, suturing full-thickness procedures being the most commonly performed after both RYGB and SG. The present review aims to detail the primary bariatric endoscopic procedures currently performed in clinical practice focusing on their mechanisms of action, efficacy, and application in the clinical practice. Revisional bariatric endoscopic procedures are detailed in the review focusing on endoscopic management after RYGB and SG. 

## 2. Primary Bariatric Endoscopic Therapies

### 2.1. Restrictive Gastric Procedures

#### 2.1.1. Intragastric Balloons 

IGBs were the first endoscopic therapeutic option developed for obesity [21]. IGBs carry out their action by occupying space in the stomach, thus reducing its volume, creating a physical impediment to the ingestion of food, and slowing gastric emptying.

**Figure 1:**
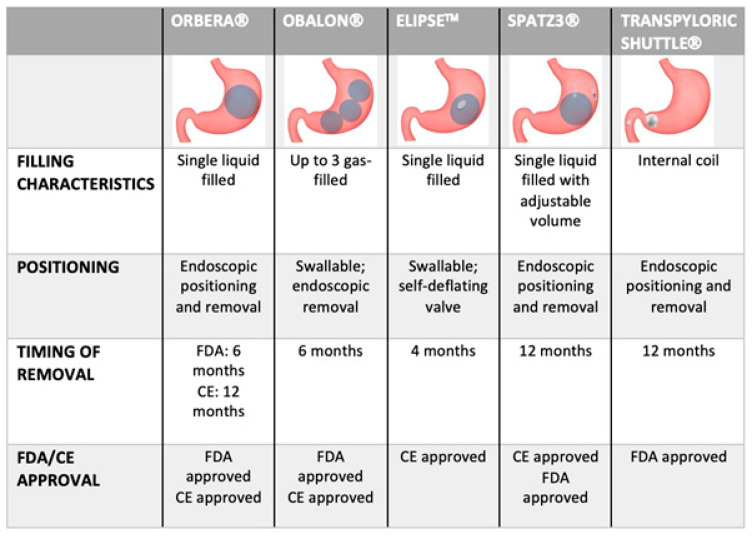
Schematic representation and characteristics of commercially available space-occupying devices.

Most of the IGBs available today have a round or oval shape made of silicone, limiting gastric mucosal injury (Figure 1). 

They are usually inflated with a fluid (saline solution, together with methylene blue) or, less frequently, with a gas, to a volume of 500–700 mL. Larger volumes lead to greater total body weight loss (TBWL), but smaller volumes are better tolerated by the patient. IGBs are generally placed endoscopically under sedation; the majority remain implanted for an average of six months and are subsequently removed endoscopically. The presence of gastric, duodenal, and esophageal ulcers, irrespective of the presence of active bleeding, a previous gastric surgery, gastric and esophageal varices, hiatal hernia >5 cm, and anticoagulant use are absolute contraindications to implantation [22].

Although the placement of an IGB is generally well tolerated, some patients may complain of adaptive symptoms or experience adverse events (AEs). The formers, such as persistent nausea, vomiting, generalized abdominal pain and/or discomfort, and reflux symptoms, are related to the space-occupying action in the gastric lumen and usually appear immediately after the insertion of the IGB and are self-limiting. However, the persistence of obstructive symptoms may require early IGB removal, which usually occurs in less than 5% of patients limiting their efficacy [23]. Serious AEs (SAE) include mucosal injury or perforation of the stomach or esophagus, gastrointestinal obstruction due to the migration of the balloon, gastric outflow obstruction, and infections due to bacterial overgrowth in the fluid filling the balloon [23,24]. A meta-analysis by Trang et al. conducted on a total of 938 patients who underwent the positioning of different types of IGBs, showed that nausea and vomiting were very frequent after an IGB positioning (63.3%; 95%CI 61.5–65.2% and 55.3%; 95%CI 53.6–57% respectively) whereas the account rate of SAEs was lower (5.2%; 95% CI 4.8–5.6%) [25].

IGBs are completely reversible, and once removed, the stomach returns to its pre-implantation condition of anatomy and functioning. This kind of reversibility allows for an application in different clinical situations. IGBs could be used as primary therapy in patients who are overweight or have mild to moderate obesity, with a target of TBWL around 10–12% [13]. A meta-analysis of nine RCTs showed that IGB implantation is superior to diet modification alone in achieving BMI loss and EWL loss [26]. However, after IGB removal, compliance with diet in the long-term period is essential in order to prevent weight regain. A recent meta-analysis reported a decrease in %TBWL to 6.9 at 18–24 month follow-up after IGB removal, indicating weight regain [27,28]. Another well-established indication is the implantation of IGB as a bridge to surgery. It is known that surgery could be challenging and associated with increased morbidity in patients with severe and very-severe obesity compared to patients with lower BMI [29,30]. For this reason, bridging therapy has been proposed for weight reduction before bariatric surgery to decrease operative difficulties and achieve better outcomes. IGBs efficacy as a bridge-to-surgery therapy in very-severe obesity patients was shown in a recent meta-analysis that reported a BMI reduction of 6.6 kg/m^2^ before surgery [31]. 

To date, three IGBs have been approved by the FDA in the US, whereas one more device is available only in Europe.

The first balloon designed in accordance with the Tarpon Springs Directives of 1987 [32], which represented the first guidelines regarding IGBs, was the Bioenterics IGB (now available as “Orbera”).

Orbera^®^ (Apollo Endosurgery, Austin, TX, USA), commercially available since 1991 and approved by the FDA in 2005 and subsequently by CE, is a single spherical silicone balloon of about 13 cm in maximum diameter. The device is positioned endoscopically. Then the balloon is inflated with saline solution to a volume of 500–700 mL. After the phase of filling, the infusion system is closed, and a self-sealing valve allows the safe release of the filling tube, which is extracted through the mouth. The balloon is inflated in the gastric fundus, and when released, it is free to float in the whole stomach. It remains in place for 6 months, and then it is removed in order to avoid mucosal injuries [33,34]. A systematic review and meta-analysis conducted by the ASGE, including 1683 patients from 17 studies, showed that Orbera^®^ achieved 11.27% of TBWL (95% CI, 8.17–14.36%) at 12 months after implantation and a significant weight loss compared to controls (+26.9% percent excess weight loss (EWL); *p*, 0.01) [35]. Another large meta-analysis by Kumar et al. [36] showed that the percentage of TBWL was 13.2% (95%CI: 12.3–14.0) at 6 months, with no differences between balloon filling volumes (400 mL vs. 700 mL). The most frequent side effects, similarly to other IGBs, are nausea and vomiting; the aforementioned meta-analysis by Trang reported a nausea and vomiting rate slightly higher than other IGBs (82%;95% CI 77–87 and 72.2; 95% CI 66.7–77.7 respectively) [25].

An Obalon^®^ (Obalon Therapeutics Inc, Carlsbad, CA, USA) balloon is compressed into a gelatinous capsule attached to a thin 2 Fr catheter. Under fluoroscopic guidance, the patient ingests the capsule, and once it reaches the stomach, the balloon is inflated with a gas (mostly nitrogen) to a maximum volume of 250 mL. Finally, the inflation catheter is removed. A maximum of 3 balloons can be placed in the stomach of a patient [37]. After a maximum of 6 months the balloons should be removed endoscopically [14,33,38]. Recently, the FDA approved the Obalon navigation system, that is, a portable console that dynamically tracks the balloon during placement using magnetic resonance and does not require X rays to confirm balloon positioning. The SMART trial, a comparative study of 387 patients between Obalon and the placebo, showed at six months a TBWL of 7.1 ± 5.3 kg in the Obalon group compared to 3.6 ± 5.1 kg in the placebo group (*p* < 0.0001) [39].

An interesting balloon is Elipse™ (Allurion, Natick, MA, USA), a swallowable balloon liquid-filled that has a self-deflating valve mechanism that allows self emptying after 4 months and spontaneous expulsion without the need for an endoscopy [40]. A study conducted on 112 patients showed a total weight loss of 10.9% at 6 months after implantation [41]. Spatz3^®^ (Spatz, Fort Lauderdale, FL, USA) is a balloon filled with 400–700 mL of saline solution that requires endoscopic positioning; its peculiarity is the guaranteed duration of 12 months and the possibility to increase or reduce the volume of the balloon endoscopically in case of low efficacy or intolerance respectively [42]. A recent study by Fittipaldi-Fernandez et al. showed that after Spatz3^®^ placement, mean BMI decreased from 39.5 to 32.8 kg/m^2^ (*p* < 0.0001) [43]. Another randomized trial on 288 patients evaluated the efficacy of Spatz3^®^ placement for 8 months compared with diet modification alone. Filling volumes of IGB were modified during the implantation period according to its efficacy or patients’ tolerance. TBWL was 15% (95%CI 13.9–16.1) in the IGB group compared to 3.3% (95%CI 2–4.6%) in the control group (*p* < 0.0001). At 6 months from IGB removal, 74% of patients had weight loss maintenance satisfying the endpoint (>50%) [44]. Spatz3^®^ received FDA approval in 2021, whereas Elipse™ is approved only by CE.

ReShape Duo was an approved integrated dual balloon system (ReShape Medical, Inc, San Clemente, CA, USA) that consisted of two liquid-filled silicone spheres joined by a flexible silicone shaft [45]. However, at the end of 2018, ReShape Medical was purchased by Apollo Endosurgery (Apollo Endosurgery, Austin, TX, USA), which decided to stop the production of ReShape Duo and provide Orbera only.

The recent Spanish Intragastric Balloon Consensus provided practical recommendations for IGB implantation. The minimum BMI for balloon implantation is 25 kg/m^2^ after failed clinical treatment. Regarding patients with a BMI of 25–30 kg/m^2^, a 6-month fluid-filled balloon is preferred, whereas in patients with BMI > 40 kg/m^2^, a 12-month fluid-filled balloon is preferred (consensus > 75%). For patients with a BMI of 30–40 kg/m^2^, which is the most common indication for IGBs implantation, there is a lower consensus to use a 12-month fluid-filled balloon [23]. 

#### 2.1.2. Transpyloric Shuttle

Transpyloric shuttle (TPS) (BARONova Inc, San Carlos, CA, USA) is an FDA-approved device since 2019, indicated for obese patients with a body mass index (BMI) of 30 to 40 kg/m^2^. It consists of a large spherical bulb attached to a smaller cylindrical bulb through a catheter. TPS is endoscopically released and fully assembled in the stomach, and designed to remain in place for up to 12 months. After the release, peristalsis carries the smaller sphere beyond the pylorus, causing intermittent gastric outlet obstruction and, thus, delaying gastric emptying [46]. To date, few studies have evaluated its safety and effectiveness. In a recent sham-controlled study conducted by Rothstein et al., 270 patients were randomized to a 12-month treatment or sham procedure; the preliminary abstract-based data showed a 30.9% excess weight loss (EWL) in the treatment group vs. 9.8% EWL in controls (*p* < 0.0001). Early device removal was required in 10.3% of patients, and the SAE rate was 2.5% [47].

#### 2.1.3. Endoscopic Gastroplasties

Endoscopic gastroplasty is proposed as the endoluminal equivalent of surgical SG. FDA- and CE-approved dedicated devices (the OverStitch™ by Apollo Endosurgery, Austin, TX and Per-Oral Incisionless Operating Platform– IOP by USGI Medical, San Clemente, CA, USA) are used in order to plicate the gastric wall and to reduce the volume of the stomach inducing an early sensation of fullness. The procedure is performed under general anesthesia. 

Endoscopic sleeve gastroplasty (ESG) was first described in 2013 by Abu Dayyeh [48] and is performed with the OverStich™ system. A suturing device is mounted on the tip of a dual-channel endoscope and is equipped with a curved needle guide that allows for either interrupted or continuous sutures. This system also uses an instrument to grasp the tissue called a “tissue helix” that is inserted through one of the channels [49].

On the other hand, primary obesity surgery endoluminal (POSE) is performed with the IOP system. It is a more complex device, equipped with a 54 Fr handle-controlled tube (TransPort^®^) able to be maneuvered in four directions and with four channels that house specialized instruments for grasping tissue folds (g-Lix™ and g-Prox EZ^®^) for positioning tissue anchors (g-Cath EZ™), and for lumen visualization with an ultra-slim endoscope [50,51].

**Figure 2:**
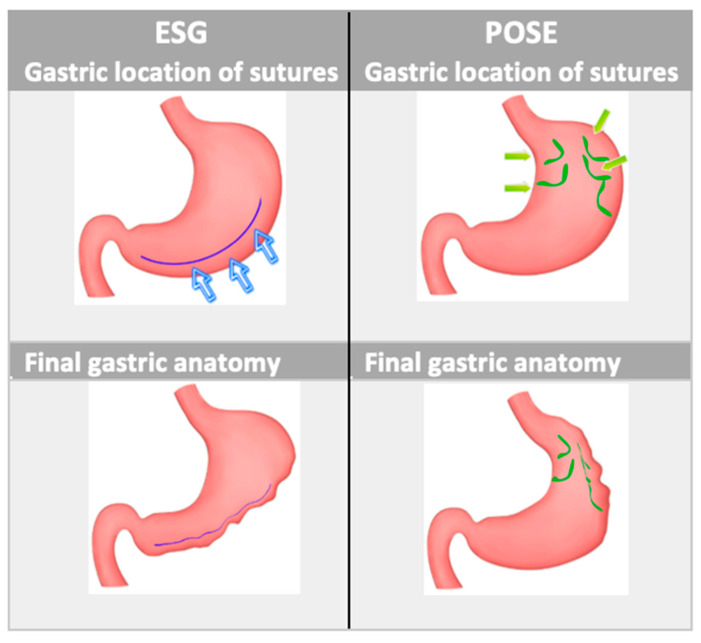
Schematic representation of endoscopic gastroplasties: on the left, an ESG procedure performed with Overstich™, and on the right, a POSE procedure performed with IOP. Blu and green arrows indicate position of endoscopic sutures during ESG and during POSE respectively. ESG, endoscopic sleeve gastroplasty; POSE, primary obesity surgery endoluminal; IOP, Incisionless Operating Platform.

The two procedures, as shown in Figure 2, result in two different types of gastroplasty. During ESG, the plication generally starts from the incisura angularis and then rises towards the gastric body along the greater curvature, shortening the distance between the anterior and posterior walls. 

During the POSE procedure, the anchor points are usually positioned in the fundus by creating eight to nine plications; three to four plications are usually placed in the distal body near the mouth of the antrum opposite to the incisura angularis in order to disrupt the gastric antral mill. A meta-analysis conducted by Gys et al., including 2475 patients, compared ESG vs. POSE, showing that both procedures were effective and safe, with only 25 patients experiencing major AEs but without the occurrence of deaths; however, ESG seemed superior in terms of EWL (68.3% vs. 44.9% respectively at 12 months) [52].

An interesting meta-analysis by Mohan et al. [53] compared ESG with surgery, showing that ESG had lower TBWL at 12 months compared to surgery (17% vs. 30.5%, *p* = 0.001) but a significantly lower rate of AEs (2.9% vs. 11.8%, *p* = 0.001).

In their recent systematic review and meta-analyses, Hedjoudje et al. confirmed the promising results of ESG in the long-term period, showing a mean TBWL of 15.1% (95% CI, 14.3–16.0) at six months, of 16.5% (95% CI, 15.2–17.8) at 12 months, and 17.2% (95% CI, 14.6–19.7) at 18–24 months. The procedure was also safe, with a pooled rate of severe AEs of 2.2% (95% CI, 1.6–3.1%), including pain or nausea requiring hospitalization (1.1%), upper gastrointestinal bleeding (0.6%), and peri-gastric leak or fluid collection (0.5%) [54]. A recent RCT comparing ESG with diet modifications alone (MERIT study) in patients with obesity grade 1 and 2 showed significant TBWL (13.6%) at 52 weeks in the ESG group that was maintained at 104 weeks in almost 70% of patients [55]. Few data are also present in the literature about the application of ESG in super obese patients with contraindications to surgery [56] or as a bridge-to-surgery procedure [57].

More recently, different plication technique variants have been proposed by leading centers in order to optimize the efficacy of gastroplasty [58,59,60]. The group of Lopez-Nava et al. modified the POSE technique performing the plication in the gastric body in order to alter its motility (POSE-2) [58]. Their preliminary data on 73 patients have been encouraging, showing a TBWL of 15.7% at 6 months with no AEs. Further studies are needed in order to confirm the lower rate of AEs and their efficacy compared to other techniques.

Regarding the comparison between endoscopic gastroplasty and IGBs, several studies showed that IGBs are less effective than gastroplasty. A retrospective study showed a significantly lower percentage of TBWL at 6 months (15.0 vs. 19.5%) and 12 months (13.9% vs. 21.3%) and higher AEs rates (17% vs. 5.2%, *p* < 0.048) in IGBs group compared to endoscopic gastroplasty [61]. Interestingly, a higher AEs rate was found in the IGBs group. A recent meta-analysis confirmed that IGB might be inferior to EGS in terms of WL; IGB-related AEs were lower than those of EGS [27]. 

Endoscopic gastroplasty also appears to have a significant metabolic effect: an observational study by Sharaiha et al. showed that ESG induces favorable changes in metabolism and obesity complications. In this study, patients had a significant reduction in liver enzymes, HbA1c, triglyceride level, and systolic blood pressure at 12-month follow-up following ESG [62]. The MERIT study also showed a significant improvement in metabolic comorbidities in patients who underwent ESG compared to controls [55].

### 2.2. Aspiration Therapy

Aspiration therapy was a promising technique performed with AspireAssist^®^ System (Aspire Bariatrics, Inc. King of Prussia, PA, USA) for class II-III obesity approved by the FDA. It consists of a customized percutaneous endoscopic gastrostomy tube associated with an external device, which aspirates approximately 30% of the gastric content after a meal. In a multicenter American study, 171 patients were randomized into two groups (aspiration + lifestyle changes vs. lifestyle modification alone) and followed up for 4 years. The average BMI of patients at the beginning of this study was 41.6 ± 4.5 kg/m^2^. After 12 months, the average BMI in the aspiration group (82 patients) was 34.1 ± 5.4 kg/m^2^ with a %TWL decreased of 18.3 ± 8.0%, and after 48 months, the %TWL in 58 patients was 18.7% [63]. Unfortunately, on February 2022, the AspireAssist system was withdrawn from the market due to financial reasons [64].

## 3. Endoscopic Malabsorptive Interventions

### 3.1. Duodenal Mucosal Resurfacing

Duodenal mucosal resurfacing (DMR), better known by the brand name Revita^®^ (Fractyl Health, Lexington, MA USA), is a catheter-based technique that hydrothermally ablates the duodenal mucosa, primarily designed for the treatment of T2DM. The Revita^®^ DMR is introduced through the mouth into the duodenum and placed distally to the Ampulla of Vater over a guidewire using fluoroscopic guidance; once in place, the gastroscope is re-inserted in order to control the procedure. The 2 cm balloon catheter is designed to isolate the mucosa from the deeper layers of the duodenum by the injection of saline solution and then hydrothermally ablate the mucosa between the Ampulla of Vater and the Treitz ligament [65]. 

The theoretical basis of this technique derives from the assumption that the duodenal mucosa of T2DM patients is abnormally hypertrophied with a higher concentration of enteroendocrine cells leading to a higher secretion of GIP that produces insulin hypersecretion and insulin resistance. 

In the first human study (proof-of-concept) [65], 39 patients with T2DM were treated with DMR, with a baseline HbA1c of 9.6% ± 1.4 and a BMI of 30.8 ± 3.5 kg. HbA1c was reduced by 1.2% at 6 months in the full cohort (*p* < 0.001), with more effects in the long segment cohort, also accompanied by a modest weight reduction of 3.9 ± 0.5 kg at 3 months (*p* < 0.001) and 2.5 ± 0.1 kg at 6 months (*p* < 0.05). In this study, no perforation, gastrointestinal bleeding, or evidence of malabsorption occurred, but three patients developed duodenal stenosis.

A recent randomized, double-blind, sham-controlled trial (REVITA-2) included 56 patients from Europe and Brazil treated with Revita^®^ DMR and 52 with a sham procedure [66]. The primary endpoint was the change of HbA1c, and one of the secondary endpoints was the change of weight at 24 weeks compared to baseline. Statistical analysis of the groups was stratified by region due to the statistical differences between sham groups. In particular, in the European group treated with DMR (N = 39), the median reduction of HbA1c from baseline at 24 weeks was –6.6 mmol/mol (2.8%), compared with –3.3 mmol/mol (2.5%) in the sham procedure group (*p* = 0.033).

Considering the secondary endpoint, in the European DMR group, the median weight reduction at 24 weeks was −2.4 kg, significantly greater than the −1.4 kg observed in the sham group (*p* = 0.012). 

The device appeared to be safe: the most common AEs in the first 30 days were transient and mild abdominal pain (17.9%) and hypoglycemia (7.7%). No severe complications occurred in the European group, whereas one jejunal perforation repaired surgically was observed in the Brazilian group. There were no episodes of pancreatitis nor infection in either group, and follow-up endoscopies revealed complete healing of the duodenal mucosa. Revita^®^ is currently for investigational use only.

### 3.2. Duodenal-Jejunal Bypass

EndoBarrier^®^ (GI Dynamics, Boston, MA USA) is a 60 cm thin and flexible Teflon-coated tube that works as a duodenal-jejunal bypass liner. The device is placed endoscopically and anchored to the duodenal bulb like a self-expanding metal stent for up to 12 months. It is a malabsorptive device that works mimicking RYGB: it brings the food to the proximal jejunum, bypassing the duodenum, preventing contact with the mucosa and the absorption of food. EndoBarrier^®^, should not interact with the Ampulla of Vater, allowing pancreatic and biliary fluids to flow down outside the tube and meet the chyme at the end of the tube [67]. Different RCTs comparing EndoBarrier^®^ to lifestyle modifications were performed around 2010, showing interesting results in both weight loss and diabetes control [35]. The first systematic review and meta-analyses conducted by Rohde et al., including five RCTs with 235 subjects and ten observational studies with 211 subjects, showed that EndoBarrier^®^ was associated with significant differences in body weight (−5.1 kg; 95% CI −7.3, −3.0) and EWL (12.6%; 95% CI 9.0, 16.2) compared to diet modification alone, even if the reduction of HbA1c and fasting plasma glucose did not reach statistical significance [68]. A subsequent systematic review reported greater reductions in both HbA1c (1.3% or 13.3 mmol/mol) and weight (TBWL 18.9%) [69]. However, the spread of EndoBarrier^®^ into clinical practice was deeply affected by the removal of the CE mark and suspension of FDA approval during the pivotal trial (ENDO trial) [70] owing to the high incidence of liver abscess in the cohort of Endobarrier patients. However, after a review of the relevant safety data, the FDA and Institutional Review Board approved the new STEP-1 pivotal trial (NCT04101669) on Endobarrier in the United States in February 2019, with study closure expected in 2025 [71]. 

Recently, a multicenter RCT was performed in the UK on 170 adults with obesity and uncontrolled T2DM [72]. The study did not achieve the primary outcome because the reduction of HbA1c ≥ 20% at 12 months did not differ in the two groups [DJB 54.6% (n = 30) vs. control 55.2% (n = 32); odds ratio (OR) 0.93, 95% CI 0.44–2.0; *p* = 0.85]. However, the study demonstrated the superiority of DJB over intensive medical care alone to achieve weight loss at 12 months: 24% (n = 16) of patients achieved ≥ 15% weight loss in the DJB group compared to 4% (n = 2) in the control group (OR 8.3, 95% CI: 1.8–39; *p* = 0.007). 

The weakness of these results was confirmed in a recent meta-analysis, where the DJBL group showed superior excess weight loss (+ 11.4% [+ 7.75 to + 15.03%], *p* < 0.00001), higher decrease in HbA1c compared to the control group (−2.73 ± 0.5 vs. −1.73 ± 0.4, *p* = 0.0001), and a SAEs rate of 19.7%, criteria that were not sufficient to reach the ASGE threshold for the treatment of obesity (i.e., ≥25% excess weight loss (%EWL) compared to the control group and ≤5% SAEs) [73].

### 3.3. Gastroduodenojejunal Bypass 

Gastroduodenojejunal Bypass (GDJB) (Endo Bypass System, ValenTx, Maple Grove, MN, USA) is a 120 cm fluoropolymer sleeve anchored in the region of gastroesophageal junction that leads the food into the small bowel with a combined endoscopic/laparoscopic procedure. This liner induces weight loss by mimicking an RYGB for 12 months until removal. In the first study evaluating the safety and efficacy of GDJB, 13 patients (mean BMI 42 kg/m^2^) were prospectively enrolled for a 1-year trial [74]. Ten patients concluded the study period, although four patients had a partial cuff detachment. At 12 months, the average EWL was 35.9% and 54% in the fully-attached subgroup. The sleeve was safe and well tolerated, with no esophageal leak, ulceration, or pancreatitis observed during the follow-up period. Five of the six patients that elapsed a period of one year with a fully attached device were followed up, and they had maintained an average EWL of 30% at the 14-month post-explant control (26 months from the beginning of the study). No further studies were performed with this type of device. Endo Bypass System is not yet approved by the FDA and CE for sale.

### 3.4. The Incisionless Magnetic Anastomotic System

The Incisionless Magnetic Anastomotic System (IMAS) (GI Windows, Westwood, MA, USA) is a novel technique that creates an anastomosis without bowel incision by using simultaneously two octagonal magnets. They are delivered into the proximal jejunum and terminal ileum with a simultaneous enteroscopy and colonoscopy (even if laparoscopic assistance is necessary), creating an anastomosis by causing local tissue necrosis. When the anastomosis is completely created, the magnets usually fall into the stool within 2 weeks. The anastomosis diverts nutrients and bile acids into the ileum, causing malabsorption.

The pilot study enrolled ten patients with an average BMI of 41 kg/m^2^ [75]. After 12 months, the average TBWL was 14.6% with an EWL% of 40.2%; moreover, in the subgroup of diabetes patients, IMAS induced a reduction of HbA1c by 1.9% and by 1.0% for prediabetic patients. All patients experienced diarrhea in the following days, and four patients had frequent diarrhea. The anastomosis is not reversible, and no data are available about long-term malabsorption consequences.

## 4. Revisional Endoscopy after Bariatric Surgery

Primary bariatric surgery, both restrictive and malabsorptive, has a notable efficacy, inducing up to 20–50% of TBWL. However, up to 1/3 of patients undergoing bariatric surgery have subsequent weight regain or insufficient weight loss. In the case of ESG, weight regain could be higher, involving up to 75% of patients [76]. Different definitions of weight regain are available, but the most commonly used definitions in clinical practice and in the literature are (1) BMI ≥ 35 kg/m^2^ after successful weight loss; (2) an increase >25% EWL from nadir; (3) an increase >10 kg from nadir; and (4) maintaining <20% of TWL [77]. The causes of weight regain are multifactorial, with the main contributing factors being a lack of lifestyle changes (i.e., sedentary life) and a lack of change in eating habits. Post-surgical anatomical factors also play an important role in weight regain. In the case of RYGB, dilation of the gastro-jejunal anastomosis (GJA) is sometimes observed, which induces a reduction of satiety and contributes to weight regain. Similarly, in ESG, dilation of the gastric remnant can be observed, leading to pre-surgical treatment satiety perception. In the past, lifestyle corrections and re-surgery were the only options available in case of weight regain. However, they carried insufficient results or significant (15–50%) post-surgical comorbidities, respectively [15]. Endoscopic therapies are attractive, as they are more effective than lifestyle modification and are associated with lower AEs rates compared with revision bariatric surgery. Below are detailed all the available endoscopic techniques applied for revision after bariatric surgery.

### 4.1. Endoscopic Revision after Roux-en-Y Gastric Bypass

Argon plasma coagulation (APC) applied at the level of GJA is a commonly available and easy-to-use treatment in the cases of endoscopic revision after RYGB. The rationale of this type of treatment is that mucosal healing of GJA mucosa after APC application induces an increase of tissue fibrosis and reduction of GJA size, thereby reducing the amount of food passing through the anastomosis [78]. More recently, a similar rationale has been exploited with the cryoablation technique commonly used for Barrett’s mucosal eradication. Similarly to APC, a cryoablation balloon is applied at the level of GJA and could also be extended at the gastric pouch leading to a fibrotic stricture and consequently to a reduction of both size of GJA and pouch [79]. APC treatment requires more than one endoscopic session (usually every 2–3 months) in order to reach the target of GJA diameter of 8–10 mm, whereas cryoablation is a one-session technique. One limitation of cryoablation is that the balloon requires a gastric pouch length of at least 4 cm. One retrospective study showed that high-dose APC (70–80 W) compared to low-dose APC (45–55 W) induced higher TBWL (10% vs. 5%) in the long term [80]. A recent RCT demonstrated the efficacy of APC over the standard multidisciplinary diet approach in terms of significant weight loss (9.73 vs. + 1.38) and improvement of quality of life after bariatric surgery [81]. Similar results have been shown for cryoablation in the only available study: significant reduction of the GJA diameter (24 to 17 mm, *p* < 0.001), pouch length (5 to 4 cm, *p* < 0.05), and short-term TBWL (8% at two months). However, it is necessary to highlight that in this study, three severe Aes (13.6%) occurred. One was GJA stenosis requiring endoscopic dilation, and two involved bleeding from the treated area [79].

**Figure 3:**
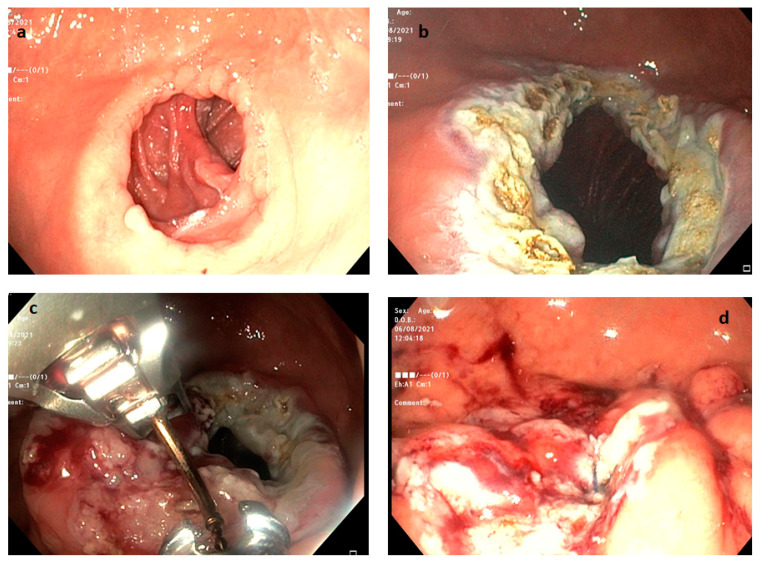
Endoscopic steps of the transoral outlet reduction endoscopic (TORe) procedure: (**a**) visualization of the dilated GJA; (**b**) APC application at the level of GJA; (**c**) suturing performance with OverStich™ system; and (**d**) final GJA at the end of the procedure. GJA, gastrojejunal anastomosis; APC, argon plasma coagulation.

Another technique aimed at reducing the GJA size is the full-thickness suture, specifically named transoral outlet reduction endoscopically (TORe). The procedure consists of the application of interrupted or purse-string stitches with the OverStich™ system at the level of GJA in order to reduce its size. Several studies have demonstrated the efficacy of TORe, showing a TBWL at one year between 6.6% and 8.6% [15,82,83]. The procedure is usually safe, and the most common AE is stricture which occurs in 3.3–4.8% of patients, whereas only one episode of severe bleeding has been described in a previous study [83]. A modified technique consists of the application of APC at the level of GJA before the execution of the TORe (Figure 3). 

A recent meta-analysis showed that APC-TORe is more effective than TORe alone, showing a TBWL at 12 months of 9.5% (5.7–13.2) vs. 5.8% (4.3–7.1) [83]. 

Older studies described the application of sodium morrhuate at the level of GJA for the treatment of weight regain after bariatric surgery. However, subsequent studies demonstrated its inferiority when compared to other endoscopic procedures (i.e., APC and suturing) [84]. A promising technique is the application of over-the-scope clips on two sides of the GJA, which showed a significant decrease in BMI levels in one study [85].

### 4.2. Endoscopic Revision after Sleeve Gastrectomy 

Weight regains after SG is a major challenge considering that it afflicts up to 2/3 of patients. RYGB or repeating SG are the most common surgical solutions for this relevant clinical problem. However, revisional surgery carries a high rate of AEs, described in up to 15% of patients [86]. In 2017 the first series of five patients was published, showing the efficacy of Endosleeve after SG (R-EndoSleeve) [87]. The procedure is analogous to the one performed as a primary intervention: each suture is started at the anterior wall of the sleeve, with subsequent bites progressing along the “greater curvature/staple line” and to the more proximal posterior wall. Approximately 6–10 bites per suture are performed. More recently, the group of de Moura et al. published a larger retrospective series of 34 patients that successfully underwent Endosleeve after SG. The study showed technical success in 100% of cases with a TBWL > 10% at 1 year in 82.4% of patients [76]. The efficacy of R-Endosleeve was confirmed in the recent prospective study by Maselli DB et al. on 82 patients who experienced significant weight regain after SG. The performance of R-endosleeve allowed a TBWL of 15.7% ± 7.6% at 12 months; ≥15% TBWL was achieved in 52.4% of patients at 12 months. The procedure was also safe with only one moderate AE (stricture at the level of the gastroesophageal junction) that was resolved with one session of endoscopic dilation [88].

Table 1 summarizes the results of the most relevant studies present in the literature for all primary and revisional bariatric endoscopic procedures.

## 5. Conclusions

Bariatric patients are a complex category that requires a multidisciplinary approach. Different professional figures, such as nutritionists, psychologists, and internal medicine physicians, are involved in the initial evaluation of obese patients and their complications [89]. These figures are essential in order to start the first-line therapeutic approach consisting of dietary regimens and changes in lifestyle habits. However, in severely obese patients and in those not compliant with conservative regimens, more invasive options such as surgery or endoscopy are offered in order to maximize clinical results and improve quality of life [3]. Both types of procedures are offered to obese patients that have failed diet modifications and have no associated psychiatric conditions [90]. Bariatric surgery largely demonstrated efficacy in terms of weight loss in the long-term period [7,10]. In the last decades, several endoscopic options have been developed as a less invasive alternative for the primary treatment of obesity. The availability of different options allows personalized treatment for different clinical situations. IGBs are the most versatile endoscopic procedure that does not alter gastric anatomy. This important feature allows its use both as a primary therapy in patients with mild obesity and also in overweight patients in order to improve metabolic complications [23,28]. Another application is the bridge-to-surgery implantation in severe and very-severe obesity patients in order to reduce intra and post-surgical complications [31]. Different types of IGBs are available in clinical practice. However, there is no evidence of superiority in the efficacy of a brand compared to another one. The general rule is that the higher BMI, the higher volumes and longer times of implantation are required [23]. 

**Figure 4:**
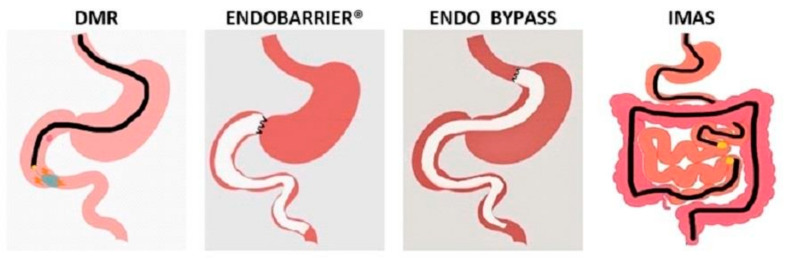
Schematic representation of the endoscopic malabsorptive procedures. IMAS, Incisionless Magnetic Anastomotic System; DMR, duodenal mucosal resurfacing.

Endoscopic gastroplasty obtained with both ESG and POSE is a well-established treatment that maximizes efficacy in type 1 and 2 obesity. Endoscopic gastroplasty leads to a lower percentage of TBWL than bariatric surgery but is superior to IGBs in inducing persistent weight loss, and therefore, patients could be offered the option to refuse surgical treatments [54,55,91]. More recently, other endoscopic procedures that have mainly malabsorptive and metabolic-modulating actions have been developed in order to induce weight loss and also to improve glycemic control (Figure 4). Revita^®^ DMR demonstrated encouraging results in pivotal trials, showing a significant reduction of HbA1c compared to controls but associated with a modest weight loss [66]. This type of procedure could be relevant to overweight and mild to moderately obese patients with difficult glycemic control in order to prevent diabetic complications. However, DMR is not yet approved by the bridFDA. An endobarrier is an ideal theoretical device that mimics malabsorptive surgical procedures without altering the anatomy. Initial results were encouraging in terms of weight loss and glycemic control [68]. However, its approval process was troublesome for the occurrence of SAE (i.e., hepatic abscess), which led to the suspension of the pivotal trial [70]. After the review of safety data, a new pivotal trial was started, and data about efficacy and safety will be available in 2–3 years [71]. IMAS and GDJB were both developed with malabsorptive intentions, but evidence of their efficacy is still limited to a few studies [74,75]. 

The choice between the different endoscopic bariatric procedures and bariatric surgery should be guided according to each specific clinical situation and should therefore be taken according to multidisciplinary decisions aiming to reduce the potentially life-threatening complications of obesity and to improve quality of life and life expectancy of these patients. 

Considering patients who have already undergone bariatric surgery, insufficient results are reported in up to one third of cases. Endoscopy has proved efficacy in revisional therapies, allowing for the optimization of surgical results and the avoidance of unacceptable rates of complications of redo-surgery [15]. In the case of RYGB, the application of APC at the GJA in association with TORe has proven the most effective results [83]. Other procedures, such as cryoablation and the application of OTSC at the level of GJA, have shown promising results in recent studies [79,85]. In case of failed SG, bariatric endoscopy could offer the R-EndoSleeve as an optimizing therapy [76,88].

In conclusion, bariatric endoscopy is a valid alternative to surgery for the treatment of obesity, offering different options that can be tailored to the patient guaranteeing good clinical results if associated with adequate diet control. As a minimally invasive technique, bariatric endoscopy may also limit the burden of AEs. The continuous evolution of endoscopic bariatric procedures has also led to the development of techniques that have the potential role of improving metabolic alterations.

## Figures and Tables

**Table 1:** Summary of the most relevant studies on the efficacy and safety of primary and revisional endoscopic therapies.

			Type of Study	Number of Patients	Comparator Group	Weight Loss Outcome	AEs Outcome
Primary endoscopic therapies	Space occupying devices	Orbera^®^	Meta-analysis [35]	1638 (17 studies)	None	EWL 25.44 (95% CI, 21.47–29.4) at 1 year	33.7% pain and nausea 1.4% migration 0.1% gastric perforation
		Obalon^®^	Double-blind RCT with sham group [39]	387	Lifestyle therapy	TBWL 7.1 ± 5.3 vs. 3.6 ± 5.1 kg	0.4% SAEs (one bleeding and one balloon deflation
		Elipse™	Prospective observational [41]	112	None	TBWL 7.9% at 1 year	51% nausea and vomiting 0% SAEs [25] *
		Spatz3^®^	RCT [44]	288	Lifestyle therapy	TBWL 15.0% vs. 3.3% 32 weeks at 32 wks (*p* < 0.0001)	Seven SAEs (7%). No deaths
		Transpyloric Shuttle^®^	Observational [46]	20	None	EWL 41% at 6 months	10% early removal for gastric ulceration
	Endoscopic gastroplasties	ESG	Meta-analysis [53]	1815 (8 studies on ESG) 2179 (7 studies on LSG)	Laparoscopic sleeve gastrectomy	TBWL 17.1% vs. 30.5% (ESG vs. LSG) at 1 year	Overall AEs 2.9% (95% CI 1.8–4.4) vs. 11.8% (95% CI 8.4–16.4)
		POSE	Meta-analysis [52]	465 (5 studies on POSE) and 1717 (8 studies on ESG)	ESG	EWL 44.9 ± 2.1% vs. 68.3 ± 3.8% (POSE vs. ESG) at 1 year	4 SAEs for POSE (3 bleeding and 1 hepatic abscess)
	Endoscopic malabsoptive procedures	DMR	RCT [66]	108	Sham procedure	HbA1c reduction from 8.5 ± 0.7% to 7.5 ± 0.8%	None
		EndoBarrier^®^	RCT [70]	80	Conventional medical therapy	TBWL 9.7% vs. 2.1% at 1 year	19 (39%) SAEs (11 re-intervention)
		Endo Bypass System	Prospective observational [74]	13	None	EWL was 35.9% at 1 year	None
		IMAS	Prospective observational [75]	10	None	TBWL was 14.6%; EWL% 40.2% at 1 year	Diarrhea
Revisional Endoscopic therapies	APC		RCT with sham group [81]	42	Diet	−9.73 kg vs. + 1.38 kg at 6 months	None in 1 year follow-up period
	Cryoablation		Retrospective series [79]	22	None	TBWL 8.1% at 8 weeks	13.6% (one stenosis and 2 bleeding)
	TORe		Meta-analysis [82]	850 (13 studies)	None	TBWL 8.55% at 1 year	Total 11.4% ± 10.11 Severe 0.57% ± 1.35
	TORe + APC		Meta-analysis [83]	1625 (16 studies)	TORe	TBWL at 12 months 9.5% vs. 5.8%	Strictures in 4.8% of patients
	Sodium morrhuate		Prospective comparative [84]	43	TORe	TBWL 2.7% ±5.5 vs. 10.4% ± 2.2	N/A
	OTSC		Observational [85]	94	None	BMI drop from 32.8 (±1.9) to 27.4 (±3.8) at 1 year	Two stenoses requiring endoscopic dilation
	R-Endosleeve		Prospective [88]	82	None	TBWL 15.7% (±7.6%) at 1 year	One moderate adverse event

IGBs, intragastric balloons; ESG, endoscopic sleeve gastroplasty; POSE, primary obesity surgical endoluminal; APC, argon plasma coagulation; TORe, transoral outlet reduction endoscopically; OTSC, over-the-scope clip; LSG, laparoscopic sleeve gastrectomy; TBWL, total body weight loss; EWL, excess of weight loss; AEs, adverse events; SAEs, severe adverse events. * Data extracted from a meta-analysis on two studies with Elipse on 42 patients.

## Data Availability

Not applicable.
